# Energy-Efficient Routing Protocol for Selecting Relay Nodes in Underwater Sensor Networks Based on Fuzzy Analytical Hierarchy Process

**DOI:** 10.3390/s22228930

**Published:** 2022-11-18

**Authors:** Jitander Kumar Pabani, Miguel-Ángel Luque-Nieto, Waheeduddin Hyder, Alfonso Ariza

**Affiliations:** 1 Department of Telecommunication Engineering, Dawood University of Engineering and Technology, Karachi 74800, Pakistan; 2 Institute of Oceanic Engineering Research, University of Malaga, 29010 Málaga, Spain; 3 Department of Computer Science, Faculty of Engineering and Computational Science, Millennium Institute of Technology and Entrepreneurship (MITE), No 10, Sector 47, Korangi Creek Road, Karachi 75190, Pakistan

**Keywords:** fuzzy analytical hierarchy process, UWSNs, routing protocols

## Abstract

The use of underwater sensor networks (UWSNs) offers great advantages in many automatic observation services such as water monitoring (ocean, sea, etc.) and registering of geological events (landslides, earthquakes). However, UWSNs have many more limitations than terrestrial sensor networks (smaller bandwidth, higher delays, etc.) with new requirements such as low power consumption by nodes or being able to select appropriate routes in a dynamic topology due to water currents and movements. To cope with these problems, the use of a routing protocol is very important. In this paper we propose a routing technique that adapts to changes in the network topology, avoiding multiple retransmissions that would affect its overall performance. This protocol is energy-efficient and is implemented using a fuzzy analytical hierarchical process (FAHP) under multi-criteria decision making (MCDM) to make an intelligent routing decision based on objectives, criteria and alternatives. To select the next node on the route, several comparison matrices are used: number of hops, distances to the sink node, and number of neighbors. The results show that the proposed setup behaves similarly to other existing underwater sensor network routing schemes using fuzzy schemes such as SPRINT.

## 1. Introduction

Underwater wireless sensor networks (UWSNs) are a technological solution used today for multiple applications, such as ocean monitoring (seismic, pollution, oil spills), early warning detection (tsunamis), or surveillance operations (underwater target location, surveillance of a country’s aquatic territory). Nowadays, the scope of UWSNs is growing in applications such as deep sea monitoring, i.e., the network SeaWeb (a UWSN implemented by means of digital signal processing telesonar acoustic modems to help in connecting fixed sensors with the mobile sensors), or in energy saving programs such as Project Natick from Microsoft (underwater datacenter cooled by the ocean to save electricity costs) [1]. However, the performance of UWSNs is affected by a wide range of problems and challenges: changes in network topology, low bandwidth, high ocean noise and interferences, and high propagation delays, among others. In addition, there are many limitations in these networks, such as low reliability, reduced network throughput, and inefficient energy consumption [2].

UWSNs were deployed initially based on the concept of terrestrial WSNs (TWSNs), but due to the differences cited, the technologies would become different. Moreover, TWSNs use radio frequency as a communication medium and employ traditional protocols such as Transmission Control Protocol (TCP), which is not suitable for underwater communications [3].

The multiple constraints which affect the performance of UWSNs should be considered in the design of the network. These restrictions include attenuation, noise, and delays due to changes in water temperature, pressure, salinity, oceanic currents, etc. UWSNs are composed of sensor devices used for sensing, processing, and communication. For efficient communication, in the initial UWSN setup, several different functions are performed: data gathering, data scheduling, forwarding the data, data aggregation, localization of the nodes, and data fusion. During these phases, the most important requirement is to save the battery energy because of the limited power capabilities of sensor nodes, which collect and forward the data to the sink node for processing [4]. Based on the challenges faced by UWSNs, it is obvious that they consume more energy than TWSNs. Thus, to address these issues, the use of routing protocols is effective [5]. 

Generally, these routing techniques are classified into two types: proactive and reactive. The proactive routing protocol is a table-driven protocol, and the forwarding delay is short because the node already knows where to forward the packet consulting the table stored. All nodes store the route information and change it accordingly as the network changes. On the contrary, the reactive routing protocol finds the route on demand by sending many request packets, which causes large delays. UWSN routing can take advantage of opportunistic routing which is an emerging technique because of its remarkable capability to enhance network throughput, reliability, and energy consumption [6].

The performance of UWSNs can be improved by taking advantage of data reception at neighboring relay nodes and their cooperation in forwarding the data to the next hop. Since the sensor nodes have limited energy, it is impossible for the nodes to forward the packets by utilizing only one-hop routing; therefore, multihop routing is preferred. The problem is when source nodes send the data to all their neighbor nodes overhearing the packets within the transmission range, owing to the propagation nature of wireless communication. Thus, selecting an efficient forwarding relay is the main task [7,8]. Besides many other challenges in UWSNs, the packet forwarding or relaying through a node with energy efficiency and low end-to-end (E2E) delay will be crucial, while the second best relay node will be selected among all the remaining candidate nodes [9].

A way to establish a relay forwarding scheme is by setting the priority among the nodes: a node with the highest priority will be selected for packet forwarding to the next hop. Several investigations have been conducted on the selection of forwarding nodes, which include the weight methods as described in the SPRINT protocol [10]. Information residing in the routing tables allows assigning the forwarding node based on those with lower distance and lower hop counts from the sink, as in [11]. One problem with some of these techniques is that some useful input parameters, such as the received signal strength indicator (RSSI) as a distance measure, or the number of hops, number of neighbors, and residual energy of a node, are not considered while designing the protocol. 

To address these issues, in this work, we introduce a forwarding relay mechanism based on two tasks: (i) selecting the best forwarding node using fuzzy decision jointly with a weight method and (ii) using the time-division multiple access (TDMA) scheme among nodes to avoid collisions, reducing delay. In order to implement the novel fuzzy decision presented in this work, the SPRINT protocol [10] is used, but the decision process is changed to select the forwarding node to include FAHP. FAHP is based on pairwise comparisons between nodes to assign a score value (named relative importance) of a node over the other under a specific criterion. So, three novel strategies are introduced (details in Section 4.2) for determining the relative importance of the comparisons, one for every criterion considered in this work: distance to the gateway, number of hops, and number of neighbors. After that, the relative importance values are arranged in three matrices (one for each criterion) before FAHP can be applied. Lastly, a final score is calculated for every candidate node, and the highest score node is selected as the best option to be the next node in the path.

The rest of the manuscript has the following structure: Section 2 presents the related works, Section 3 explains the system model employed for UWSNs, Section 4 gives all of the details of the proposed scheme based on FAHP, and Section 5 shows the results by simulations carried out in comparison with the SPRINT [10] protocol. Finally, Section 6 presents a brief discussion and remarks on the main conclusions obtained in this work.

## 2. Related Work

In the literature, several approximations for the establishment of the initial path in WSNs can be found. Our proposed classification for strategies related to the scope of this work is as follows: (1) those using fuzzy decisions, (2) those using hierarchy processes, and (3) those using other specific routing techniques. 

The use of fuzzy decisions in routing protocols has received growing interest in recent years. One of the classic applications is to select the cluster head (CH) node in a cluster-based network topology, using multiple criteria in the assignment of that role mainly to increase the network lifetime. In this area, we have decision schemes such as the fuzzy technique for order of preference by similarity to ideal solution (Fuzzy-TOPSIS) [12]; LEACH fuzzy clustering (LEACH-FC) [13]; data gathering protocol in unequal clustered WSNs utilizing fuzzy decisions (DGUCF) [14]; multiple-attribute decision making (MADM) [15] using criteria such as residual energy, distance from the base station, and number of neighbors; energy-efficient distributed clustering algorithm based on fuzzy scheme (EEDCF) [16] to overcome uneven load on the network and select CH using the fuzzy Takagi–Sugeno–Kang (TSK) model; adaptive network based on fuzzy inference system (ANFIS) [17] which employs a fuzzy neural network; or using the density of nodes [18] jointly with the Mamdani method of fuzzy inference for selecting the CH.

Another typical application for fuzzy decisions is the selection of an efficient routing path using multihop links (node-by-node hops), such as in the relay node selection scheme based on fuzzy inference algorithms (RNSFIA) [19]. In RNSFIA, a fuzzy inference algorithm is used to select the relay node, where the criteria used for the decision are distance between nodes, priority on residual energy, and degree of communication. Compared to MOD-LEACH [20], RNSFIA has a higher throughput and network lifetime. Another example for efficient routing with fuzzy decisions is multi-criteria decision making (MCDM) [21], where weights and criteria such as hop count, packet transmission frequency, and residual energy are used.

A second block of strategies (hierarchy processes) is based on multiple comparisons under different criteria. Examples include the analytical hierarchy process (AHP) [22], which selects the relay node in body area networks (WBANs) using weights among several candidate nodes; AHP MCDM [23], which uses a two-phase clustering scheme that includes finding the location of the nodes by using sink position and criteria such as the number of neighbors, centrality, and residual energy; or the analytical network process (ANP) [24] based on MCDM which selects the best CH node using criteria such as initial and residual energy, energy consumption rate, average energy of the network, and distance of a node from CH. Others (e.g., [25]) consider both ANP and AHP using a fuzzy scheme for solving the CH selection in a cluster network. In the UWSN area, various methods are considered to achieve energy efficiency. We have used input parameters such as hop count, distance to the sink, and number of neighbors using the FAHP MCDM strategy and obtained better results compared to some of the existing research techniques. FAHP is almost identical to AHP except for the conversion of verbal appreciation into the numeric scale. AHP indicates the relative importance of criteria in MCDM, being preferable in qualitative judgments, and cannot accept fuzzy numbers as input. Thus, fuzzy AHP (FAHP) was introduced because AHP lacks the benefits of managing vagueness in judgment. For example, a decision maker cannot make exact judgment between number 4 and 6 instead of using exactly the number 5. However, fuzzy numbers are the way to integrate such imprecision, giving benefits for FAHP compared to AHP schemes.

The last family of routing techniques considered here is out of the scope of the two cited before. Usually, a specific solution is proposed for the path’s creation through the network. For example, in the distributed energy-efficient zonal relay node-based secure routing protocol (DEZMSR) [26], a relay node is selected based on zone radius by means of a division in two kinds of nodes, namely zonal and district relay nodes; other examples include using load balance [27], using comparison rules such as the Cauchy inequation [28] between energy used and routing distance, employing a specific hierarchy in the cluster deployment [29], and using a weighted product model (WPM) [30], among many others.

In conclusion, the effectiveness of fuzzy algorithms for selecting the CH node in clusters has been proved. However, routing in an ad hoc topology requires first creating the paths between every node and the sink node (on the surface) with an unknown location of the nodes. On the other hand, complex multi-criteria decision problems can be managed efficiently by a standard formulation using hierarchy processes such as AHP. To our knowledge, there is no routing protocol using FAHP in the underwater environment for a random network topology that does not use clusters as hierarchy. In this work, we show that is possible to apply AHP including fuzzy decisions (FAHP) to the problem of the initial path creation after deployment of a random-topology UWSN. From our point of view, this is a new alternative for performing node forwarding selection in UWSNs.

## 3. System Model

**Figure 1:**
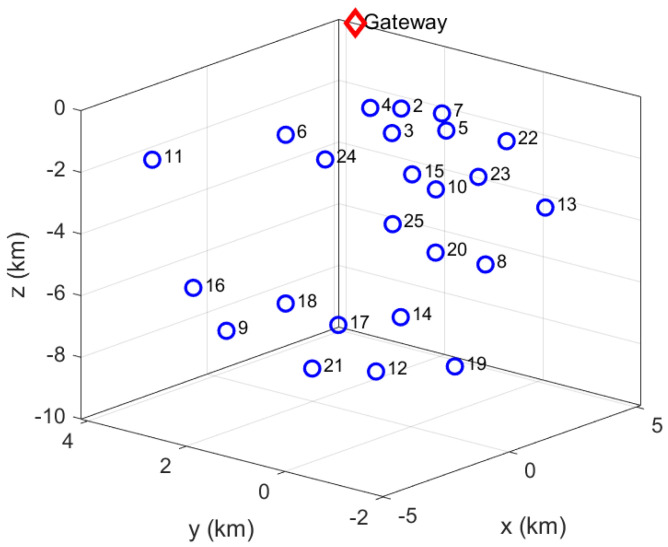
Random node deployment in 3D UWSNs. Data: 25 nodes (including gateway on surface), deployment zone: 10 × 6 × 10 km, minimum separation between nodes: 1 km.

The UWSN routing protocols usually involve four phases: network deployment, neighbor discovery, relay node selection, and communication phase. The scope of this work is limited to relay node selection by using decision making. Figure 1 shows a node deployment model (3D UWSN), where nodes are located randomly at various locations and depths, except for the gateway (GW) node that resides on the surface (random location too). After the deployment task, we can consider for analysis purposes that nodes will remain static (anchored to the seafloor or fixed to a buoy) and be placed far enough to prevent sending the same packet from multiple nodes to the sink or avoid undesired overlaps (minimum distance between two adjacent nodes or neighbor nodes).

In the creation of a path from a node to the gateway, the next forward node selection has been performed by taking into account input parameters such as distance between nodes, number of hops to reach the GW, number of neighbor nodes, and the transmission range by applying a fuzzy process (based on FAHP technique) that will be explained later. As a result, the last node of the path chooses as next relay node the node having the highest score (weights in FAHP) among the rest of candidate nodes.

### Energy Consumption Model

The sensor nodes are usually powered through batteries, and it is inconvenient to replace or recharge them when they are depleted, so designing an efficient routing scheme is a key challenge. The energy used to send the data from one node to another node over distance d is given by

(1) $$E_{d}=E_{t}\left(d\right)+E_{r}\left(d\right)$$

where $E_{t}\left(d\right)$ is the transmission energy and $E_{r}\left(d\right)$ is the reception energy. Both energies are affected by certain parameters given in the following equations:

(2) $$E_{t}\left(d\right)=k\left(E_{\mathrm{elec}\;}+\varepsilon_{\mathrm{amp}\;}\right)+P_{t}\frac{k}{\alpha \cdot B\left(d\right)}$$

(3) $$E_{r}\left(d\right)=k\left(E_{\mathrm{elec}\;}+E_{DA}\right)+P_{r}\frac{k}{\alpha \cdot B\left(d\right)}$$

where $P_{t}$ and $P_{r}$ are the transmission and reception powers, respectively; *k* indicates the packet length; $E_{\mathrm{elec}\;}$ is the electronic energy consumed; $\varepsilon_{\mathrm{amp}\;}$ is the amplifier energy; $E_{DA}$ is the energy consumed throughout the data aggregation process; α is the modulation efficiency; and B(d) is the available bandwidth.

## 4. Relay Scheme based on FAHP

The proposed protocol uses a fuzzy analytical hierarchy process (FAHP) to select the best relay node among several alternatives. These alternatives are selected based on three input parameters: number of hops, number of neighbors, and distance to the gateway.

### 4.1. Fuzzy Analytical Hierarchy Process

Fuzzy AHP is a technique used to solve many complex problems, and it is efficient in decision-making problems, which usually are complicated to manage. In AHP, the decision is made based on priorities, and the best decision is selected among the alternatives. AHP consists of three levels: the main goal, the evaluation criteria, and the alternatives. During a decision-making process, the alternatives are compared with each other, which helps in the selection of the best choice. Pairwise AHP is helpful in reducing complicated decisions and becomes helpful for decision makers, allowing them to reduce decision biases. In AHP, every criterion has a weight assigned that indicates its importance. The proposed criteria are then applied to the alternatives, assigning a priority to each of them [31] to select the best candidate. The objective of AHP is to select the alternative (node) that best describes the set of criteria, calculating the weights of each criterion.

Fuzzy pairwise comparison is applied to criteria and alternatives through linguistic variables which are represented by real numbers named triangular fuzzy numbers (TFNs). A membership function is the linear representation of the TFNs. We will use a triangular shape function which can be defined as follows:

(4) $$\mu \left(x\right)=\{\begin{array}{ll} \;0 & x<l\;\\ \frac{x-l}{m-l} & l\;\leq \;x<m\\ \frac{u-x}{u-m} & m\;\leq \;x<u\;\\ \;0 & x\;\geq \;u\; \end{array}$$

where l,  m,  u, are the TFNs: l is the lower bound value, m is the middle value, and u is the upper bound value. Many operations can be performed on TFNs, and in our case, we will employ three of them: addition, multiplication, and reciprocal. These operations can be introduced by the following equations:

(5) $$\left(l_{1},m_{1},u_{1}\right)⊕\left(l_{2},m_{2},u_{2}\right)=\left(l_{1}+,l_{2},m_{1}+m_{2},u_{1}+u_{2}\right)$$

(6) $$\left(l_{1},m_{1},u_{1}\right)⊗\left(l_{2},m_{2},u_{2}\right)=\left(l_{1}*l_{2},m_{1}*m_{2},u_{1}*u_{2}\right)\;$$

(7) $${\left(l_{1},\;m_{1},u_{1}\right)}^{-1}=\left(\frac{1}{u_{1}},\frac{1}{m_{1}},\frac{1}{l_{1}}\right)\;$$

It can be noted in (7) that in the reciprocal TFN, an increasing order of the components is needed to fulfill the definition of a membership triangular function.

In this work, we will use both the Chang [32] and Buckley [33] methods to calculate the final weights for selecting the best alternative. The steps of applying FAHP are given in the next subsections.

#### 4.1.1. Criteria Selection

**Figure 2:**
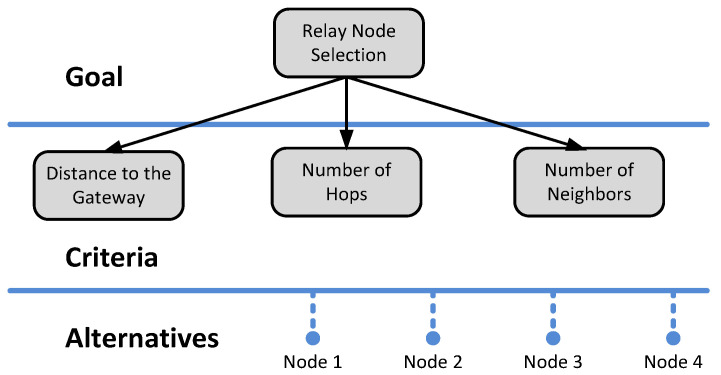
Hierarchy diagram for FAHP scheme applied to select the best relay node of four candidates.

The problem of selecting the relay node can be represented by a three-level diagram as shown in Figure 2. We will consider here three criteria: distance to the gateway, number of hops, and number of neighbors. Additionally, we choose to limit the alternatives to four (or fewer, depending on the number of candidate nodes).

In a four-level scheme, FAHP can contain sub-criteria under the criteria level. However, in the case of interest, due to the ad hoc network being random, we do not have more criteria relevant than those mentioned, because the topology is unknown until the paths are created.

#### 4.1.2. Pairwise Comparison Matrix

The next step is to create a comparison matrix between every pair of criteria, which means setting how important a criterion is compared to another one. For example, if a candidate node (i.e., within the transmission range of the node running FAHP) is far from the gateway (which is bad for routing due to high delay) but has many neighbors (high reliability in reaching the gateway), which criteria will be more important? The answer is a pairwise matrix that establishes the level of importance between two criteria. This is the starting point of FAHP, similar to establishing priorities between the set of criteria. 

Firstly, we should consider a range of values to measure importance. Usually, this importance value follows the Saaty scale [33] (see Table 1), ranging from *equal significance* (value 1) to *extreme significance* (value 9).

Secondly, the pairwise comparison matrix T for the *k* decision maker’s preference is given by

(8) $$T^{k}=\left[\begin{array}{cccc} m_{11}^{k} & m_{12}^{k} & \cdots & m_{1n}^{k}\\ \cdots & \cdots & \cdots & \cdots \\ m_{n1}^{k} & m_{n2}^{k} & \cdots & m_{nn}^{k} \end{array}\right]$$

where the element $m_{ij}^{k}$ stands for the *k-*preference of *i*-criterion over *j*-criterion, using TFNs.

#### 4.1.3. Average Pairwise Comparison Matrix

In the case of *K* multiple decisions (K ≥ 1), it is necessary to average all of them, using

(9) $${\tilde{m}}_{ij}=\frac{1}{K}\overset{K}{\underset{k=1}{\sum }}m_{ij}^{k}$$

yielding an average comparison matrix $\tilde{T}$, expressed as

(10) $$\tilde{T}=\left[\begin{array}{ccc} {\tilde{m}}_{11} & \ldots & {\tilde{m}}_{1n}\\ \vdots & \ddots & \vdots \\ {\tilde{m}}_{n1} & \cdots & {\tilde{m}}_{nn} \end{array}\right]$$

#### 4.1.4. Geometric Means

By using the geometric means method of Buckley [33], the TFN values of every criterion in the fuzzy pairwise comparison can be obtained by the following equation:

(11) g˜i=(∏j=1nm˜ij)1n, i=1,2,…, n

where ${\tilde{g}}_{i}$ is the geometric mean TFN value for the *i-*criterion.

#### 4.1.5. Fuzzy Weights of Criteria

The calculation of the weights for each *i-*criterion can be summarized in the following operation

(12) $${\tilde{w}}_{i}={\tilde{g}}_{i}⊗{\left({\tilde{g}}_{1}⊕{\tilde{g}}_{2}⊕\;\cdots \;⊕{\tilde{g}}_{n}\right)}^{-1}$$

where addition (⊕), multiplication (⊗), and reciprocal (${\left(\;\right)}^{-1}$) operations over TFNs have been previously defined in (5)–(7). Note that the fuzzy weight for the *i-*criterion ${\tilde{w}}_{i}$ resultant is a TFN as well, so it can be expressed in its components of the membership function as

(13) $${\tilde{w}}_{i}=\left(lw_{i},mw_{i},uw_{i}\right)$$

#### 4.1.6. Real Normalized Weights

At this point, we have a TFN weight for every *i-*criterion (${\tilde{w}}_{i}$). In order to obtain a single value for every TFN (${\tilde{w}}_{i}$), is necessary to perform the defuzzification process. In our case, we employ the well-known center of area method [33], given by

(14) $$q_{i}=\frac{l_{w_{i}}+m_{w_{i}}+u_{w_{i}}}{3}$$

where $q_{i}$ stands for the final real weight for the *i-*criterion.

In order to use a relative quantity among all the criteria set, is necessary to normalize every weight by using the following relation:

(15) $$l_{i}=\frac{q_{i}}{\sum_{k=1}^{n}q_{k}}$$

where $l_{i}$ is the normalized weight of the *i-*criterion.

### 4.2. Measurement of Relative Importance

Before applying the FAHP technique presented, we need to establish how to compare two nodes under the three criteria considered and give a value (the relative importance) as a result on the Saaty scale (Table 1). 

#### 4.2.1. Criterion 1: Distance to the Sink

**Figure 3:**
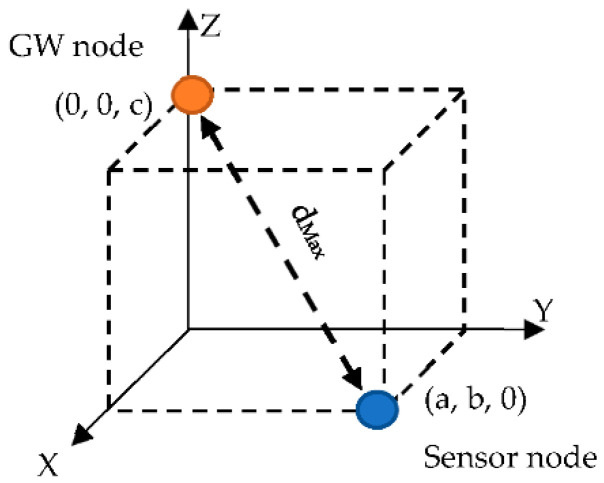
Maximum distance in a 3D box.

With the objective of obtaining the relative importance between two nodes on the Saaty scale, an initial estimation value for the longest possible direct route is marked in Figure 3, which would be that between a node located in the bottom corner opposite to the upper corner in diagonal, where the GW would be located (on the surface). This maximum distance of the direct longest route (no hops, only theoretical) is given by

(16) $$d_{Max}=\sqrt{{\left(a-0\right)}^{2}+{\left(0-b\right)}^{2}+{\left(0-c\right)}^{2}}=\sqrt{a^{2}+b^{2}+c^{2}}$$

Of course, there could be longer routes once they are created due to the multiple hops to reach the GW, but this value can be updated as long as it is known by the network (i.e., the route has reached the final node, the GW).

The final objective of the comparison now is to set the relative importance between two nodes for constructing the pairwise matrix that is necessary to apply FAHP among the alternatives (not between every pair of criteria, which has been set by $T^{k}$ in (8)).

**Figure 4:**
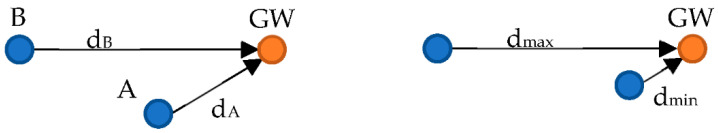
Distances (**left**) and constraints (**right**) for applying the distance criterion.

In Figure 4, we introduce a situation to introduce the problem of comparing the relative importance of distance to the gateway node. We have two nodes *A* and *B* with different distances *d_A_* and *d_B_*, respectively. Additionally, we can assume two restrictions in the deployment zone: a minimum (*d_min_*) and maximum (*d_max_*) distance (to the gateway) that a node can have. The problem is determining how to grade the importance on the Saaty scale proposed (a value in the range [1,9]) when a node is closer to or further from the gateway than another one.

In order to compare how good the distance of node *A* to the gateway is compared to that of node *B* to the gateway, the difference between the distances can be firstly considered, and then a linear normalization can be used to give a value on the Saaty scale (within [1, 9]). For doing that, we can take into account the limit situations, i.e., the best case and the worst case. Assuming that $d_{B}>d_{A}$ (*A* is closer than *B*), both cases are defined by the difference in distances:

(17) $$\left(\mathrm{Best}\;\mathrm{case}:)\;\;\;\Delta_{b}=d_{B}-d_{A}\;/\{d_{B}=d_{max},\;d_{A}=d_{min}\right\}=d_{max}-d_{min}$$

(18) $$\left(\mathrm{Worst}\;\mathrm{case}:)\;\;\;\Delta_{w\;}=d_{B}-d_{A}/\{d_{B}=d_{A}+\delta \;\right\}=\delta$$

where $\Delta_{b}$ and $\Delta_{w}$ stand for the best and worst distance differences, respectively. The parameter δ is a threshold defining what is considered the same distance in a practical way. In order to have nine equal space intervals in the domain of difference in distances (δ, $\Delta_{b}$), a new interval $\Delta_{S,d}$ (*S* subindex comes from Saaty) is defined:

(19) $$\Delta_{S,d}=\frac{d_{max}-d_{min}}{9}$$

Finally, in order to obtain a single natural value of the importance of $d_{A}$ vs. $d_{B}$, it only remains to employ the following expression, where ⌈·⌉ is the ceil function (round toward positive infinity):

(20) $$I_{AB}=\{\begin{array}{c} \begin{array}{l} \;\;\;\;\;\;\lceil \frac{d_{B}-d_{A}}{\Delta_{S,d}}\rceil ,\;\;\;\;\;\;\;\;if\;d_{B}>d_{A}\;\;\;\;\;\;\;\;\;\;\;\;\;\;\;\\ \;\;\;\;\;\;\;\;\;\;\;\;1,\;\;\;\;\;\;\;\;\;\;\;if\;\left|d_{B}-d_{A}\right|\;\leq \;\delta \end{array} \end{array}\;\;$$

where *I_AB_* is the importance level for distance criterion on the Saaty scale (see Table 1) of node *A* vs. node *B*. Note that $I_{AB}\in \left[1,9\right]$. In case of $d_{A}>d_{B}$, the comparison is similar but in the opposite way: it would be the comparison of the *B* node with the *A* node. In that case, the reciprocal TFN value should be used in the pairwise comparison matrix for node *A* vs. node *B*.

**Figure 5:**
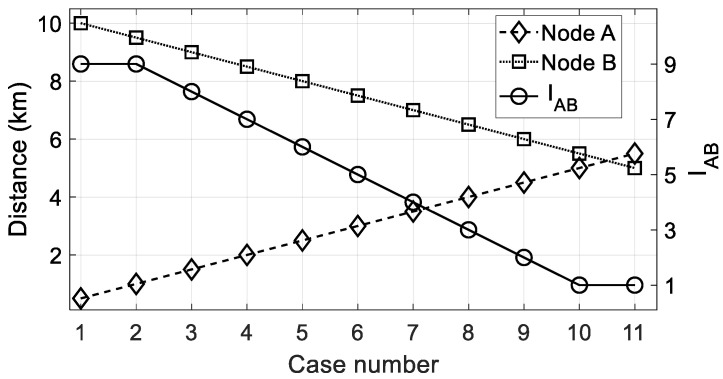
Importance value *I_AB_* (circles) when nodes *A* and *B* are compared under the distance criterion. Data: *d_max_* = 10 km, *d_min_* = 0.5 km, δ = 1.

As an example, Figure 5 shows the importance level $I_{AB}$ calculated by (20) for various locations of two nodes *A* and *B*. While one is approaching the gateway (node *B*) from the maximum distance, another one is going away (node *A*) from the minimum distance to the gateway.

#### 4.2.2. Criterion 2: Number of Hops

Following similar steps regarding the distance criterion, the number of hops is another important measure to compare candidate nodes to be selected for a routing path. The objective is clear: inside a set of several nodes (candidates), a pairwise comparison matrix must be constructed, where every value of importance for a pair of nodes (*I_AB_*) in the matrix should be on the Saaty scale (within the interval [1, 9]).

In order to set the scale of the problem, a starting point is to estimate the limits. In this sense, we can use the latter two parameters: the minimum (*d_min_*) and maximum (*d_max_*) distance to the gateway that a node can have. Assuming two nodes *A* and *B* with $d_{A}$ and $d_{B}$ distances to the gateway ($d_{B}>d_{A}$ i.e., *A* is closer than *B* to the gateway), we can consider two scenarios for the best and worst cases for estimating the *N_h_* (number of hops) as follows:

(21) $$(\mathrm{Best}\;\mathrm{case}:)\;\;\;\left\{\;d_{A}=d_{min},\;d_{B}=d_{max}\right\}\;🡺\;N_{h,A}\;=1,\;N_{h,B}=\left\lceil \frac{d_{max}}{d_{min}}\right\rceil$$

(22) $$\left(\mathrm{Worst}\;\mathrm{case}:)\;\;\;\{\;d_{B}-d_{A}=d<\delta \;\right\}\;🡺\;N_{h,A}=N_{h,B}\;\in [1,\lceil \frac{d}{d_{min}}\rceil \;]$$

where the parameter δ has the same meaning as in distance criterion: a threshold to define what is considered as the same distance in a practical way, and $N_{h,A}\;and\;N_{h,B}$ are the number of hops from every node to reach the gateway. In order to have nine equal intervals in the domain of the number of hops (1, $\left\lceil \frac{d_{max}}{d_{min}}\right\rceil$), a new interval $\Delta_{S,h}$ is defined:

(23) ΔS,h  =dmaxdmin−19

When evaluating the comparison between nodes *A* and *B*, the importance *I_AB_* in the best case would have a value of 9 on the Saaty scale, meaning that is very convenient to choose node *A* instead of node *B* under this criterion of number of hops. In the opposite case (worst), the importance *I_AB_* would have a value of 1, indicating that any node has the same number of hops, and both have the same opportunity to be selected. In a general case, the final expression for the relative importance between two nodes *A* and *B* can be given by

(24) IAB={      [dBdAΔS,h]     , if dB>dA                           1             ,if |dB−dA| ≤ δ  

where [·] stands for the nearest integer function. It can be observed again that $I_{AB}\in \left[1,\;9\right]$. Another waypoint is to compare directly the number of hops between the two nodes *A* and *B* ($N_{h,A},\;N_{h,B}$) from the limit value ($N_{h,max}=\left\lceil \frac{d_{max}}{d_{min}}\right\rceil$, $N_{h,min}=1$):

(25) $$I_{AB}=\{\begin{array}{c} \begin{array}{l} \;\;\;\;\;\;\left\lceil \frac{N_{h,B}-N_{h,A}}{\Delta_{S,NH}}\right\rceil \;\;\;\;\;,\;if\;N_{h,B}>N_{h,A}\;\;\;\;\;\;\;\;\;\;\;\;\;\;\;\\ \;\;\;\;\;\;\;\;\;\;\;\;1\;\;\;\;\;\;\;\;\;\;\;\;\;\;\;\;\;\;\;,if\;\left|N_{h,B}-N_{h,A}\right|\;\leq \;\delta \end{array} \end{array}\;\;,\;\;\;\;\;\Delta_{S,NH}\;\;=\frac{N_{h,max}-1}{9}$$

**Figure 6:**
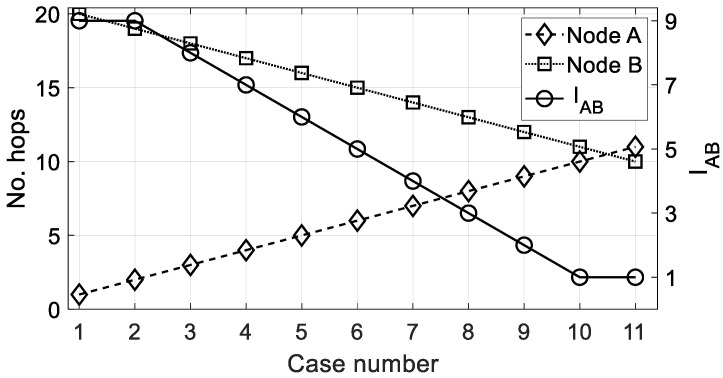
Importance value *I_AB_* (circles) when nodes *A* and *B* are compared under the number of hops criterion. Data: *d_max_* = 10 km, *d_min_* = 0.5 km, δ = 1.

As usual, in the case of $N_{h,A}>N_{h,B}$, the value of $I_{BA}$ would be the inverse of the equivalent $I_{AB}$. A similar example to that given for the distance criterion is shown in Figure 6 to prove the good behavior of (25).

#### 4.2.3. Criterion 3: Number of Neighbors

As done with the two previous criteria, distance and number of hops, the numbers of neighbors between two candidate nodes are compared, and a final value should be obtained within the Saaty scale [1, 9] to build the corresponding pairwise matrix.

In a random network, is impossible to know a priori what the maximum number of neighbors of a node is. However, we can approximate the problem to be in an intermediate situation bounded by the best and the worst topologies. 

**Figure 7:**
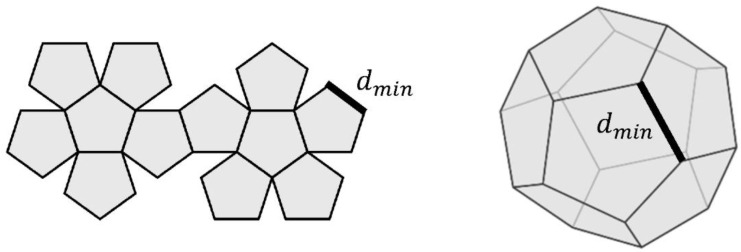
Dodecahedron: sided (**left**) and polyhedral (**right**) shape.

The best topology we can imagine is a dense and regular mesh of nodes, where all of them are at the distance *d_min_*, imposed by the deployment conditions for the network. When more nodes are included, a node in the center of a sphere of radius *d_Tx_* (transmission range) will have a greater number of neighbors. Due to the problem being 3D, it is necessary to think of filling this sphere (radius *d_Tx_*) with a regular structure of nodes. The solution is to use regular polyhedrons, which can be inscribed in a sphere and keep the regularity. That is, the nodes (vertices) will keep the distance *d_min_* between adjacent vertices. Among the five regular polyhedrons known (tetrahedron, cube, octahedron, dodecahedron, and icosahedron), the polyhedron with the greatest number of vertices is the dodecahedron with 20 vertices and 12 faces, shown in Figure 7.

The best topology for having the maximum number of neighbors is to fill the big sphere of radius *d_Tx_* with equal dodecahedrons of side length *d_min_*. This way, between two adjacent nodes (vertices), a distance *d_min_* is conserved and the mesh is a 3D structure. In spite of existing holes in the space when we fill a sphere with dodecahedra (the rhombic dodecahedron is the structure that tessellates the space without holes), we follow with this reasoning (simpler), and by using the volumes of the sphere and dodecahedron, the expression to obtain the total number of neighbors (nodes) $N_{n,\;max}$ can be approximated as

(26) $$V_{sphere}=4\;\pi \;d_{Tx}^{3}$$

(27) $$V_{dodecahedron}=\frac{1}{4}\left(15+7\sqrt{5}\right)d_{min}^{3}$$

(28) $$N_{n,\;max}\sim \frac{V_{sphere}}{V_{dodecahedron}}\times 20-N_{common}$$

where $N_{common}$ is the number of common vertices when a set of dodecahedra are joined together. This number is easy to obtain in a practical way, and a possible empirical solution is presented in Table 2 (it is not the only one, depending on the position selected for the new dodecahedron with respect to others). It is assumed that every added dodecahedron is set to share the greatest number of faces possible, for its nodes are nearest to the rest of the vertices already present. So, we can approximate that the new one added always shares three common faces.

**Figure 8:**
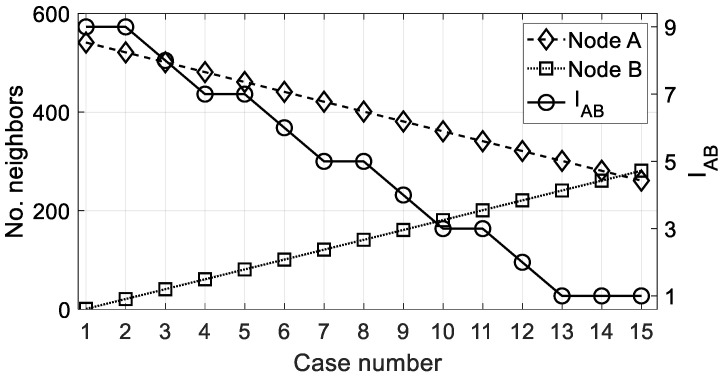
Importance value $I_{AB}$ when nodes *A* and *B* are compared under the number of neighbors criterion. Data: *d_min_* = 0.5 km, *d_Tx_* = 2 km, $N_{n,max}=550,$ $\Delta_{S,n}=61,$ δ = 5.

In the opposite direction, the worst case for node *A* having a minimum number of neighbors is that in which there exists only another node *B* inside the region included in a sphere of radius *d_Tx_* with the center being the location for node *A*. Any other situation can be assumed to be bounded into these two situations (best and worst). For this reason, to compare the number of neighbors of two nodes *A* and *B* with numbers of neighbors $N_{A}$ and $N_{B}$, respectively, and have the information value $I_{AB}\in \left[1,\;9\right]$ (Saaty scale), a suitable expression could be the following:

(29) $$I_{AB}=\{\begin{array}{c} \begin{array}{l} \;\;\;\;\;\;\left\lceil \frac{N_{n,B}-N_{n,A}}{\Delta_{S,NH}}\right\rceil \;\;,\;\;\;if\;N_{n,B}>N_{n,A}\;\;\;\;\;\;\;\;\\ \;\;\;\;\;\;\;\;\;\;\;\;1\;\;\;\;\;\;\;\;,\;\;\;if\;\left|N_{n,B}-N_{n,A}\right|\;\leq \;\delta \end{array} \end{array}\;\;\;,\;\Delta_{S,NH}\;\;=\frac{N_{n,max}-1}{9}$$

where ⌈·⌉ is the ceil function (round toward positive infinity). In Figure 8, the comparative value of the importance $I_{AB}$ is measured in a set of cases where two nodes *A* and *B* are becoming closer in number of neighbors. In the first case of the graph, $N_{n,A}=N_{n,max}$ and $N_{n,B}=1$, which is the best possible case. In this situation, $I_{AB}=9$; i.e., node *A* has the maximum chance to be selected against node *B*, considering the number of neighbors criterion. On the other hand, it can be seen that $I_{AB}$ reaches a value of 1 when both nodes have a similar number of neighbors. The values shown in Figure 8 are given in Table 3 for clarity.

### 4.3. Application of FAHP to Criteria

At this point, the FAHP will be applied specifically to our problem of selecting the best relay node among the best four alternatives (nodes). Firstly, the different criteria considered are evaluated on the Saaty scale, as shown in Table 4. These values include the reciprocal numbers calculated as their inverse values (e.g., if Distance vs. No. Hops value is 6, then No. Hops/Distance will be its reciprocal with the value 1/6). An importance value of 6, in the middle of the Saaty scale, is used for stability, although other values are possible, and the method works fine.

The next step is to apply the fuzzy technique to the relative importance of the criteria in Table 4 by introducing TFNs (4), yielding values presented in Table 5.

According to (11), we can calculate the geometric mean $\tilde{g}$ from every criterion from Table 5, yielding values in Table 6. As an example, for the distance criterion, its TFN $\tilde{g}$ extended over every criterion of the three considered will be calculated as follows:

(30) $$\tilde{g}=\left(l,\;m,u\right)=\left({\left(1\cdot 5\cdot 5\right)}^{\frac{1}{3}},\;{\left(1\cdot 6\cdot 6\right)}^{\frac{1}{3}},\;{\left(1\cdot 7\cdot 7\right)}^{\frac{1}{3}}\right)=\left(2.924,\;3.3019,\;3.6593\right)$$

Once the mean of every criterion has been obtained, is time to calculate the fuzzy weights from (12). This can be calculated by multiplying each ${\tilde{g}}_{i}$ value with the reciprocal value of the total mean, yielding the values in Table 7. For example, for the distance criterion, its TFN weight $\tilde{w}$ would be calculated as follows:

(31) $$\tilde{w}=\left(l,m,u\right)=\left(\frac{2.924}{5.12},\;\frac{3.3019}{4.6048},\;\frac{3.6593}{4.0912}\right)=\left(0.5711,\;0.7171,\;0.8944\right)$$

From Table 7, it is possible to obtain the non-fuzzy weights $q_{i}$ from (14) and their corresponding normalized values $l_{i}$ from (15), whose values are presented in Table 8. As an example, in the case of the distance criterion, its weight $q_{i}$ is calculated as (0.5711 + 0.7171 + 0.8944)/3 = 0.7275, and its corresponding normalized value is 0.7275/(0.7275 + 0.2217 + 0.0676) = 0.7155. Note that the summation of the normalized weights $l_{i}$ is equal to 1.

At this point, we have the normalized weights ($l_{i}$) corresponding to every criterion, which will be applied to every candidate node to select the best one. In order to make the final decision, is necessary to carry out a similar comparison process by means of a matrix between every pair of candidate nodes (alternatives) under every criterion considered. The result of this process is a new set of normalized weights $l_{i}^{k}$ for every alternative *i* under the criterion *k*. The whole process in an example case is given in Appendix A.

A valid definition for the final score of every alternative is given by the following rank:

(32) $$\;\;\;\;\;Rank_{i}=\overset{3}{\underset{k=1}{\sum }}l_{k}\cdot l_{i}^{k}$$

where $l_{k}$ is the local weight of criterion *k* (from Table 8) and $l_{i}^{k}$ is the weight of the criterion *k* calculated for the alternative (node) *i*. The selected alternative (node) will be the higher-ranked of the four alternatives considered.

## 5. Simulation Results

To assess the performance of FAHP, the protocol SPRINT [10] has been employed in its first version, changing the decision process of selecting the forwarding node by the multi-criteria FAHP scheme presented in this work. Moreover, to compare the results obtained with another fuzzy technique, a recent SPRINT fuzzy-based version [34] has been run under the same conditions presented in Table 9. The software used was MATLAB.

For every transmission range considered, 10 simulations were performed, each having a different random topology, and the results were averaged. The area (volume) of deployment was considered as 10 × 10 × 10 km. Although this could seem exceedingly high and deep, is convenient to have a dispersive location of the nodes in 3D, which makes it more difficult to create routes to the gateway. In addition, other authors also use the same volume for giving simulation results [35].

### 5.1. Path Length

**Figure 9:**
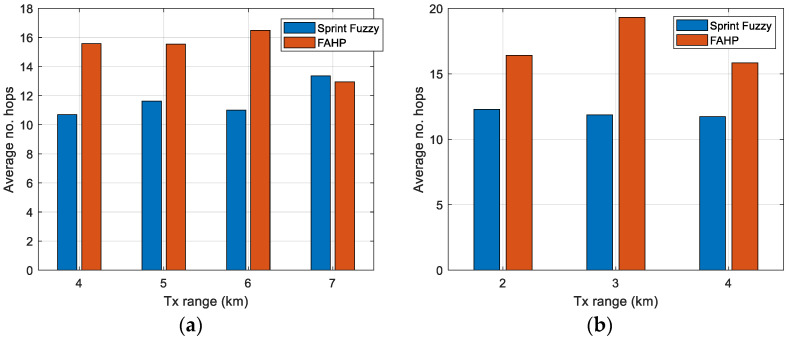
Average number of hops (left bar: SPRINT with fuzzy logic, right bar: FAHP method). Number of nodes: (**a**) 100, (**b**) 400.

One of the important parameters in the establishment of the paths for the initial random topology is the mean path length, i.e., the average number of hops of every path over all of them. Every path will end in the gateway node on the surface. The results obtained in FAHP and in SPRINT-Fuzzy [34] using different numbers of nodes (100 and 400) and different transmission ranges (4–7 km for 100 nodes, 2–4 km for 400 nodes) are shown in Figure 9.

For a better comparison, Table 10 is introduced, presenting the values obtained in the bar graph of Figure 9.

Although the conclusion is that FAHP has a worse behavior than SPRINT-Fuzzy in creating the paths, they are close (see 100 nodes, transmission range 7 km). Moreover, FAHP can admit more criteria to be taken into account for enhancing the selection. So, these results are not definitive, and they can be enhanced when including new metrics in FAHP, such as density nodes in a region and residual energy for extending lifetime.

### 5.2. Number of Collisions

A metric related to the efficiency of energy consumption is the number of collisions occurring during the phase of route creation. In this sense, a lower number of collisions means a lower energy consumption and a suitable transmission range adopted. This problem is inherent to non-guided channels such as underwater channels. In the SPRINT protocol used here, there is a TDMA-based mechanism for avoiding collisions, but due to the random position of the nodes in the network, is impossible to avoid them completely in the initial creation path phase.

**Figure 10:**
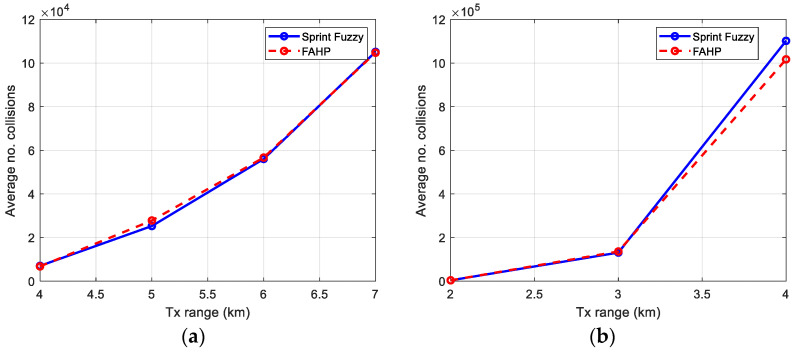
Average number of collisions. Number of nodes: (**a**) 100, (**b**) 400.

The results obtained for both methods, FAHP and SPRINT-Fuzzy, are presented in Figure 10. It can be seen that FAHP is as good as SPRINT-Fuzzy, or even better in case of a high number of nodes (e.g., 400 nodes), which is a more complex network and has more free degrees to make routes.

As previously, Table 11 is introduced, presenting the values obtained in the bar graph of Figure 10.

### 5.3. End-to-End Delay

The end-to-end (E2E) delay can be defined as the elapsed time that an outgoing packet from a node takes to reach the destination following a multihop path. In this case, this is the time taken for a packet from an underwater sensor node to reach the gateway node on the surface. The longer the route is, the higher the E2E delay is.

**Figure 11:**
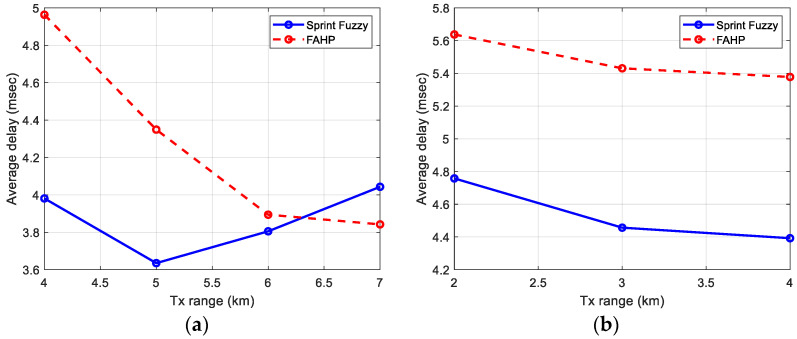
Average delay in reaching the gateway node. Number of nodes: (**a**) 100, (**b**) 400.

The results obtained by simulations for both FAHP and SPRINT-Fuzzy are presented in Figure 11. It can be noted as FAHP has a value of delay in the same order of magnitude (4–6 ms) when compared to SPRINT-Fuzzy but is a little worse in dense networks (400 nodes). The best behavior is observed when the transmission range is high, evident in Figure 11a for a transmission range of 7 km, obtaining a better delay than SPRINT-Fuzzy. This is logical due to a lower value of hops average for that specific case, shown in Figure 9a.

Table 12 presents the values obtained in the bar graph of Figure 11.

A second analysis can be performed from these results: FAHP is stable. Despite the network size increasing by a factor of 4 (from 100 to 400 nodes), the average E2E delay is kept in a 4–6 ms interval, although the transmission range is changed between 2 and 7 km.

### 5.4. Energy Consumption

In relation to energy consumption, the computation takes into account the sum of both the average energy used in transmission and the average energy used in reception for all nodes when all the paths have been created, i.e., the instant when all the nodes belong to a route that ends in the gateway node.

**Figure 12:**
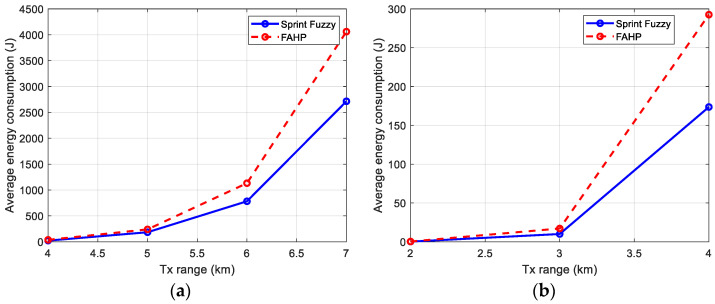
Average energy consumption. Number of nodes: (**a**) 100, (**b**) 400.

Figure 12a,b contain the average energy consumption in the network. The value is a little worse for FAHP than for SPRINT-Fuzzy in this section, but it is justified by longer routes in FAHP, which take more energy for packets to reach the gateway.

Table 13 shows the values obtained in the bar graph of Figure 12.

## 6. Discussion

After the deployment of sensors in an ad hoc UWSN, the submerged nodes have to create paths using multihop technique (from node to node) to route the packets to the gateway node on the surface. In this starting phase, parameters such as delay and throughput are unknown until all the nodes are connected by at least a route that ends in the gateway.

In that scenario, among the parameters that can be estimated are distance to the gateway (e.g., using received signal strength indicator (RSSI) in the packet), the number of hops that a node needs to reach the gateway in the partial route created, and the number of neighbors that are in the range of a node. These three parameters have been considered in FAHP to select the next node that belongs to a partial route coming from the gateway to the sea bottom. 

The FAHP scheme has been used in complex decision problems in which multiple criteria are applied to make the best selection among candidates. The problem of selecting which nodes belong to a route to have a low packet delay or energy wasted in the nodes is an interesting problem that relies on the field of FAHP.

In this work, it has been proved as with only those three parameters (distance, hops, and neighbors), the problem is solved in a random topology with efficiency similar to that of other techniques (SPRINT-Fuzzy) in time (E2E delay packet) and energy consumption. Moreover, stability can be demonstrated by the results provided when considering 400 nodes, which is a large size for this type of network. These reasons are coupled with the existence of a random topology in the initial deployment phase and the consideration that FAHP can handle more criteria than those presented here in future work, which makes the presented technique a suitable option for the routing problem in UWSNs.

## Figures and Tables

**Table 1:** Saaty scale of importance.

Importance	Definition	TFN	Reciprocal Scale TFN
1	Equal Significance	(1, 1, 1)	(1, 1, 1)
3	Moderate Significance	(2, 3, 4)	(1/4, 1/3, 1/2)
5	Strong Significance	(4, 5, 6)	(1/6, 1/5, 1/4)
7	Very Strong Significance	(6, 7, 8)	(1/8, 1/7, 1/6)
9	Extreme Significance	(9, 9, 9)	(1/9, 1/9, 1/9)
2	(Intermediate values)	(1, 2, 3)	(1/3, 1/2, 1)
4		(3, 4, 5)	(1/5, 1/4, 1/3)
6		(5, 6, 7)	(1/7, 1/6, 1/5)
8		(7, 8, 9)	(1/9, 1/8, 1/7)

**Table 2:** Common vertices in structures composed of dodecahedra.

No. Dodecahedra (*n*)	*N_common_*
2	5
3	5 + 2 × 5 = 15
4	15 + 3 × 5 = 30
5	30 + 3 × 5 = 45
More than 6	(*n* − 2) × 15

**Table 3:** Information values from Figure 8 (number of neighbors criterion).

No. case	*N_n,A_, N_n,B_*	*I_AB_*	No. case	*N_n,A_, N_n,B_*	*I_AB_*	No. case	*N_n,A_, N_n,B_*	*I_AB_*
1	541, 1	9	6	441, 101	6	11	341, 201	3
2	521, 21	9	7	421, 121	5	12	321, 221	2
3	501, 41	9	8	401, 141	5	13	301, 241	1
4	481, 61	7	9	381, 161	4	14	281, 261	1
5	461, 81	7	10	361, 181	3	15	261, 281	1

**Table 4:** Pairwise comparison matrix of criteria.

Pairwise Comparison	Distance	No. Hops	No. Neighbors
**Distance**	1	6 *	6 *
**No. Hops**	1/6	1	6 *
**No. Neighbors**	1/6	1/6	1

* 6: between 5 (strong significance) and 7 (very strong significance).

**Table 5:** Pairwise comparison matrix of criteria as TFNs.

Pairwise Comparison	Distance	No. Hops	No. Neighbors
**Distance**	(1, 1, 1)	(5, 6, 7)	(5, 6, 7)
**No. Hops**	(1/7, 1/6, 1/5)	(1, 1, 1)	(5, 6, 7)
**No. Neighbors**	(1/7, 1/6, 1/5)	(1/7, 1/6, 1/5)	(1, 1, 1)

**Table 6:** Geometric mean of criteria from (11).

Criterion	$\tilde{g}$
**Distance**	(2.924, 3.3019, 3.6593)
**No. Hops**	(0.8939, 1, 1.1187)
**No. Neighbors**	(0.2733, 0.3029, 0.3420)
**Total Mean (** $\overset{3}{\underset{i=1}{\sum }}{\tilde{g}}_{i}$ **)**	(4.0912, 4.6048, 5.12)
**Reciprocal of Total Mean**	(0.1953, 0.2171, 0.2444)

**Table 7:** Weighted matrix of criteria $\tilde{w}$ from (12).

Criterion	$\tilde{w}$
**Distance**	(0.5711, 0.7171, 0.8944)
**No. Hops**	(0.1746, 0.2172, 0.2734)
**No. Neighbors**	(0.2733, 0.3029, 0.3420)

**Table 8:** Non-fuzzy ($q_{i}$ ) and normalized ($l_{i}$ ) weights (from 14,15).

Pairwise Comparison	*q*	*l*
**Distance**	0.7275	0.7155
**No. Hops**	0.2217	0.2180
**No. Neighbors**	0.0676	0.0665

**Table 9:** Parameters of simulation.

Parameters	Value	Unit
Speed of sound	1500	m/s
Data rate	1500	bit/s
Frequency	48	kHz
Packet length	256	bits
Header length	30	bits
Transmission power	18	W
Number of nodes	100, 400	
Simulation length	10	
Minimum distance	1	km
Transmission range	2–7	km

**Table 10:** Statistical comparison for average No. hops.

No. Nodes	Tx Range (km)	Av. No. Hops	
		Sprint	FAHP (%)
100	4	10.7	15.57 (+46%)
100	5	11.62	15.54 (+33.7%)
100	6	11.01	16.49 (+49%)
100	7	13.37	12.94 (−3.2%)
400	2	12.28	16.41 (+33.6%)
400	3	11.87	19.3 (+62.6%)
400	4	11.73	15.83 (+35%)

**Table 11:** Statistical comparison for average No. collisions.

No. Nodes	Tx Range (km)	No. Collisions	
		Sprint	FAHP (%)
100	4	7041.9	6786.7 (−3.6%)
100	5	25,296.5	27,830.4 (+10%)
100	6	56,056.3	56,676.1 (+1.1%)
100	7	105,135	104,748 (−0.4%)
400	2	3680.7	3806.33 (+3.4%)
400	3	130,852	135,981 (+3.9%)
400	4	1,101,770	1,016,980 (−7.7%)

**Table 12:** Statistical comparison for E2E packet delay.

No. Nodes	Tx Range (km)	E2E Delay (ms)	
		Sprint	FAHP (%)
100	4	3.98	4.96 (+24.6%)
100	5	3.64	4.35 (+19.5%)
100	6	3.8	3.89 (+2.4%)
100	7	4.04	3.84 (−5%)
400	2	4.76	5.64 (+18.5%)
400	3	4.46	5.43 (+21.7%)
400	4	4.39	5.38 (+22.6%)

**Table 13:** Statistical comparison for average energy consumption.

No. Nodes	Tx Range (km)	Av. Energy Consumption (J)	
		Sprint	FAHP (%)
100	4	21.8	36.6 (+67.9%)
100	5	183.6	238.8 (+30.1%)
100	6	782.3	1132.14 (+44.7%)
100	7	2714.2	4060.4 (+49.6%)
400	2	0.19	0.31 (+63.2%)
400	3	10	17.1 (+71%)
400	4	173.6	292.5 (+68.5%)

## Data Availability

Not applicable.

## Appendix A

In this appendix, the weights for every alternative under the three criteria considered are calculated step by step. For using numbers more than variables, we can assume a specific case of four alternatives (nodes), where alternative 2 is the most important and alternative 3 the least.

The starting point for applying the FAHP method is to obtain the pairwise comparison matrix, presented in Table A1, Table A2 and Table A3 for every criterion. The tables have been obtained by comparing every pair of nodes of the alternative set, using the measurement explained in Section 4.2.

**Table A1:** Pairwise comparison matrix for distance criterion.

	Node 1	Node 2	Node 3	Node 4
**Node 1**	1	1/6	2	8
**Node 2**	6	1	3	7
**Node 3**	1/2	1/3	1	1/4
**Node 4**	1/8	1/7	4	1

**Table A2:** Pairwise comparison matrix for No. hops criterion.

	Node 1	Node 2	Node 3	Node 4
**Node 1**	1	1/5	2	6
**Node 2**	5	1	3	5
**Node 3**	1/2	1/3	1	1/3
**Node 4**	1/6	1/5	3	1

**Table A3:** Pairwise comparison matrix for No. neighbors criterion.

	Node 1	Node 2	Node 3	Node 4
**Node 1**	1	1/3	2	4
**Node 2**	3	1	4	3
**Node 3**	1/2	1/4	1	1/5
**Node 4**	1/4	1/3	5	1

The next step is to apply the fuzzy technique to the relative importance of the criteria, introducing TFNs (4) in Table A1, Table A2 and Table A3, yielding values presented in Table A4, Table A5 and Table A6.

**Table A4:** Pairwise comparison matrix for distance criterion as TFN.

	Node 1	Node 2	Node 3	Node 4
**Node 1**	(1, 1, 1)	(1/7, 1/6, 1/5)	(1, 2, 3)	(7, 8, 9)
**Node 2**	(5, 6, 7)	(1, 1, 1)	(2, 3, 4)	(6, 7, 8)
**Node 3**	(1/3, 1/2, 1)	(1/4, 1/3, 1/2)	(1, 1, 1)	(1/5, 1/4, 1/3)
**Node 4**	(1/9, 1/8, 1/7)	(1/8, 1/7, 1/6)	(3, 4, 5)	(1, 1, 1)

**Table A5:** Pairwise comparison matrix for No. hops criterion as TFN.

	Node 1	Node 2	Node 3	Node 4
**Node 1**	(1, 1, 1)	(1/6, 1/5, 1/4)	(1, 2, 3)	(5, 6, 7)
**Node 2**	(4, 5, 6)	(1, 1, 1)	(2, 3, 4)	(4, 5, 6)
**Node 3**	(1/3, 1/2, 1)	(1/4, 1/3, 1/2)	(1, 1, 1)	(1/4, 1/3, 1/2)
**Node 4**	(1/7, 1/6, 1/5)	(1/6, 1/5, 1/4)	(2, 3, 4)	(1, 1, 1)

**Table A6:** Pairwise comparison matrix for No. neighbors criterion as TFN.

	Node 1	Node 2	Node 3	Node 4
**Node 1**	(1, 1, 1)	(1/4, 1/3, 1/2)	(1, 2, 3)	(3, 4, 5)
**Node 2**	(2, 3, 4)	(1, 1, 1)	(3, 4, 5)	(2, 3, 4)
**Node 3**	(1/3, 1/2, 1)	(1/5, 1/4, 1/3)	(1, 1, 1)	(1/6, 1/5, 1/4)
**Node 4**	(1/5, 1/4, 1/3)	(1/4, 1/3, 1/2)	(4, 5, 6)	(1, 1, 1)

Applying (11)–(15) to the values in the Table A4, Table A5 and Table A6, it is possible to calculate the following for every node and criterion: the geometric mean $\tilde{g}$, the fuzzy weights $\tilde{w}$, the non-fuzzy weights $q_{i}$, and the normalized weights $l_{i}$. The obtained results are presented in Table A7, Table A8 and Table A9.

**Table A7:** Geometric mean ($\tilde{g}$ ) and weights ($\tilde{w}$, q, l ) for distance criterion.

	$\tilde{g}$	$\tilde{w}$	q	l
**Node 1**	(1, 1.2779, 1.5244)	(0.1511, 0.2283, 0.3318)	0.2371	0.2269
**Node 2**	(2.7832, 3.3504, 3.8687)	(0.4205, 0.5986, 0.8421)	0.6204	0.5936
**Node 3**	(0.3593, 0.4518, 0.6389)	(0.0543, 0.0807, 0.1391)	0.0914	0.0875
**Node 4**	(0.4518, 0.5170, 0.5874)	(0.0683, 0.0924, 0.1279)	0.0962	0.0920

**Table A8:** Geometric mean ($\tilde{g}$ ) and weights ($\tilde{w}$, q, l ) for No. hops criterion.

	$\tilde{g}$	$\tilde{w}$	q	l
**Node 1**	(0.9554, 1.2447, 1.5137)	(0.1504, 0.2377, 0.3621)	0.2501	0.2361
**Node 2**	(2.3784, 2.9428, 3.4641)	(0.3743, 0.5621, 0.8286)	0.5883	0.5554
**Node 3**	(0.3799, 0.4855, 0.7071)	(0.0598, 0.0927, 0.1691)	0.1072	0.1012
**Node 4**	(0.4671, 0.5623, 0.6687)	(0.0735, 0.1074, 0.1600)	0.1136	0.1073

**Table A9:** Geometric mean ($\tilde{g}$ ) and weights ($\tilde{w}$, q, l ) for No. neighbors criterion.

	$\tilde{g}$	$\tilde{w}$	q	l
**Node 1**	(0.9306, 1.2779, 1.6549)	(0.1505, 0.2593, 0.4372)	0.2823	0.2609
**Node 2**	(1.8612, 2.4495, 2.9907)	(0.3010, 0.4970, 0.7901)	0.5294	0.4893
**Node 3**	(0.3247, 0.3976, 0.5373)	(0.0525, 0.0807, 0.1419)	0.0917	0.0848
**Node 4**	(0.6687, 0.8034, 1)	(0.1082, 0.1630, 0.2642)	0.1785	0.1650

The last parameter in Table A7, Table A8 and Table A9 (normalized weights $l_{i}$) is employed for calculating the final score of every alternative: the rank (32). Using the previously calculated normalized weights for criteria in Table 8, the rank is finally calculated in Table A10. From Table A10, it can be seen that the winner is alternative 2 (node 2), which will be selected as the next forward node in the path. Note that the summation of the node ranks is unity, as was expected.

**Table A10:** Rank calculation for every node.

	Distance $\left(l_{dist}\cdot l_{node}^{dist}\right)$	No. Hops $\left(l_{hops}\cdot l_{node}^{hops}\right)$	No. Neighbors $\left(l_{neigh}\cdot l_{node}^{neigh}\right)$	Rank (∑)
**Node 1**	0.7155 × 0.2269	0.2180 × 0.2361	0.0665 × 0.2609	0.2312
**Node 2**	0.7155 × 0.5936	0.2180 × 0.5554	0.0665 × 0.4893	0.5783
**Node 3**	0.7155 × 0.0875	0.2180 × 0.1012	0.0665 × 0.0848	0.0903
**Node 4**	0.7155 × 0.0920	0.2180 × 0.1073	0.0665 × 0.1650	0.1002

## References

[1] Mahalle P.N., Shelar P.A., Shinde G.R., Dey N. (2021). The Underwater World for Digital Data Transmission.

[2] Luo J., Chen Y., Wu M., Yang Y. (2021). A Survey of Routing Protocols for Underwater Wireless Sensor Networks. IEEE Commun. Surveys Tuts..

[3] Haque K.F., Kabir K.H., Abdelgawad A. (2020). Advancement of Routing Protocols and Applications of Underwater Wireless Sensor Network (UWSN)—A Survey. J. Sens. Actuator Netw..

[4] Gupta O., Goyal N. (2021). The Evolution of Data Gathering Static and Mobility Models in Underwater Wireless Sensor Networks: A Survey. J. Ambient Intell Humaniz. Comput..

[5] Akyildiz F., Pompili D., Melodia T. (2006). Underwater Acoustic Sensor Networks: Research Challenges. Ad Hoc Networks.

[6] Awan K.M., Shah P.A., Iqbal K., Gillani S., Ahmad W., Nam Y. (2019). Underwater Wireless Sensor Networks: A Review of Recent Issues and Challenges. Wirel. Commun. Mob. Comput..

[7] Rahman M.A., Lee Y., Koo I. (2017). EECOR: An Energy-Efficient Cooperative Opportunistic Routing Protocol for Underwater Acoustic Sensor Networks. IEEE Access.

[8] Vieira L.F.M. Performance and Trade-Offs of Opportunistic Routing in Underwater Networks. Proceedings of the IEEE Wireless Communications and Networking Conference.

[9] Ghoreyshi S.M., Shahrabi A., Boutaleb T. (2016). A Novel Cooperative Opportunistic Routing Scheme for Underwater Sensor Networks. Sensors.

[10] Hyder W., Luque-Nieto M.-A., Poncela J., Otero P. (2019). Self-Organized Proactive Routing Protocol for Non-Uniformly Deployed Underwater Networks. Sensors.

[11] Hindu S.K., Hyder W., Luque-Nieto M.-A., Poncela J., Otero P. (2019). Self-Organizing and Scalable Routing Protocol (SOSRP) for Underwater Acoustic Sensor Networks. Sensors.

[12] Mustafa M. (2013). Multiple Criteria Decision-Making Based Clustering Technique for WSNs. Master’s Thesis.

[13] Lata S., Mehfuz S., Urooj S., Alrowais F. (2020). Fuzzy Clustering Algorithm for Enhancing Reliability and Network Lifetime of Wireless Sensor Networks. IEEE Access.

[14] Song X., Zhang Q., Sun W., Wei W. (2017). Energy-Efficient Data Gathering Protocol in Unequal Clustered WSN Utilizing Fuzzy Multiple Criteria Decision Making. J. Intell. Fuzzy Syst..

[15] Azad P., Sharma V. (2013). Cluster Head Selection in Wireless Sensor Networks under Fuzzy Environment. Int. Sch. Res. Not..

[16] Zhang Y., Wang J., Han D., Wu H., Zhou R. (2017). Fuzzy-Logic Based Distributed Energy-Efficient Clustering Algorithm for Wireless Sensor Networks. Sensors.

[17] Varun R.K., Gangwar R.C., Kaiwartya O., Aggarwal G. (2021). Energy-Efficient Routing Using Fuzzy Neural Network in Wireless Sensor Networks. Wirel. Commun. Mob. Comput..

[18] Adhikary D.R.D., Mallick D.K. (2017). An Energy Aware Unequal Clustering Algorithm Using Fuzzy Logic for Wireless Sensor Networks. J. ICT Res. Appl..

[19] Jain N.K., Verma A. (2019). Relay Node Selection in Wireless Sensor Network Using Fuzzy Inference System. J. Commun..

[20] Mahmood D., Javaid N., Mahmood S., Qureshi S., Memon A.M., Zaman T. MODLEACH: A Variant of LEACH for WSNs. Proceedings of the 2013 Eighth International Conference on Broadband and Wireless Computing, Communication and Applications.

[21] Bhunia S., Das B., Mukherjee N. EMCR: Routing in WSN Using Multi Criteria Decision Analysis and Entropy Weights. Proceedings of the International Conference on Internet and Distributed Computing Systems.

[22] Kim B., Cho J., Jeon S., Lee B. (2016). An AHP-Based Flexible Relay Node Selection Scheme for WBANs. Wirel. Pers. Commun..

[23] Hanifi A., Taghva M.R., Haghi R.H., Feizi K. (2018). Clustering for Reduction of Energy Consumption in Wireless Sensor Networks by AHP Method. J. Inf. Syst. Telecommun..

[24] Dalia R., Gupta R. Cluster Head Selection Technique for Improving The Network Lifetime in WSN Using ANP. Proceedings of the International Conference on Innovative Computing & Communications (ICICC).

[25] Mukherjee P., Pattnaik P.K., Al-Absi A.A., Kang D.-K. (2021). Recommended System for Cluster Head Selection in a Remote Sensor Cloud Environment Using the Fuzzy-Based Multi-Criteria Decision-Making Technique. Sustainability.

[26] Stella K., Ganesh E.N. (2018). Distributed Energy Efficient Zonal Relay Node Based Multi Path Secure Routing Protocol (DEZMSR) for Wireless Sensor Networks. J. Comput. Theor. Nanosci..

[27] Salameh H.B., Obaidat H., Al-Shamali A., Jararweh Y. (2021). A Two-level Clustering Mechanism for Energy Enhancement in Internet-of-Things-based Wireless Sensor Networks. Int. J. Commun. Syst..

[28] Li F., Wang L. (2018). Energy-Aware Routing Algorithm for Wireless Sensor Networks with Optimal Relay Detecting. Wirel. Pers. Commun..

[29] Shukla A., Tripathi S. (2020). An Effective Relay Node Selection Technique for Energy Efficient Wsn-Assisted Iot. Wirel. Pers. Commun..

[30] Das B., Bhunia S.S. Multi Criteria Routing in Wireless Sensor Network Using Weighted Product Model and Relative Rating. Proceedings of the 2015 Applications and Innovations in Mobile Computing (AIMoC).

[31] Tariq M.I., Ahmed S., Memon N.A., Tayyaba S., Ashraf M.W., Nazir M., Hussain A., Balas V.E., Balas M.M. (2020). Prioritization of Information Security Controls through Fuzzy AHP for Cloud Computing Networks and Wireless Sensor Networks. Sensors.

[32] Chang D.-Y. (1996). Applications of the Extent Analysis Method on Fuzzy AHP. Eur. J. Oper. Res..

[33] Buckley J.J. (1985). Fuzzy Hierarchical Analysis. Fuzzy Sets Syst..

[34] Pabani J.K., Luque-Nieto M.-Á., Hyder W., Otero P. (2021). Energy-Efficient Packet Forwarding Scheme Based on Fuzzy Decision-Making in Underwater Sensor Networks. Sensors.

[35] Liu J., Yu M., Wang X., Liu Y., Wei X., Cui J. (2018). RECRP: An Underwater Reliable Energy-Efficient Cross-Layer Routing Protocol. Sensors.

